# Influenza and associated co-infections in critically ill immunosuppressed patients

**DOI:** 10.1186/s13054-019-2425-6

**Published:** 2019-05-02

**Authors:** Ignacio Martin-Loeches, Virginie Lemiale, Pierce Geoghegan, Mary Aisling McMahon, Peter Pickkers, Marcio Soares, Anders Perner, Tine Sylvest Meyhoff, Ramin Brandt Bukan, Jordi Rello, Philippe R. Bauer, Andry van de Louw, Fabio Silvio Taccone, Jorge Salluh, Pleun Hemelaar, Peter Schellongowski, Katerina Rusinova, Nicolas Terzi, Sangeeta Mehta, Massimo Antonelli, Achille Kouatchet, Pål Klepstad, Miia Valkonen, Precious Pearl Landburg, Andreas Barratt-Due, Fabrice Bruneel, Frédéric Pène, Victoria Metaxa, Anne Sophie Moreau, Virginie Souppart, Gaston Burghi, Christophe Girault, Ulysses V. A. Silva, Luca Montini, Francois Barbier, Lene B. Nielsen, Benjamin Gaborit, Djamel Mokart, Sylvie Chevret, Elie Azoulay, For the Efraim investigators and the Nine-I study group

**Affiliations:** 1 Medical Intensive Care Unit, Hôpital Saint-Louis and Paris Diderot Sorbonne University, Paris, France; 2 Department of Intensive Care Medicine (710), Radboud University Medical Centre, Nijmegen, The Netherlands; 3 Department of Critical Care and Graduate Program in Translational Medicine, Programa de Pós-Graduação em Clínica Médica, D’Or Institute for Research and Education, Rio de Janeiro, Brazil; 4 Department of Intensive Care, Rigshospitalet, University of Copenhagen, Copenhagen, Denmark; 5 CIBERES, Universitat Autonòma de Barcelona, European Study Group of Infections in Critically Ill Patients (ESGCIP), Barcelona, Spain; 6 Pulmonary and Critical Care Medicine, Mayo Clinic, Rochester, MN USA; 7 Division of Pulmonary and Critical Care, Penn State University College of Medicine, Hershey, PA USA; 8 Department of Intensive Care, Hôpital Erasme, Université Libre de Bruxelles (ULB), Brussels, Belgium; 9 Department of Intensive Care Medicine, Multidisciplinary Intensive Care Research Organization (MICRO), St. James’s Hospital, Dublin, Ireland; 10 Department of Clinical Medicine, Wellcome Trust-HRB Clinical Research Facility, St. James Hospital, Trinity College, Dublin, Ireland; 11 Department of Medicine I, Medical University of Vienna, Vienna, Austria; 12 Department of Anesthesiology and Intensive Care Medicine and Institute for Medical Humanities, 1st Faculty of Medicine, Charles University in Prague and General University Hospital, Prague, Czech Republic; 13 Norwegian University of Science and Technology, Trondheim, Norway; 14 CHU Grenoble Alpes, Service de Réanimation Médicale, Faculté de Médecine de Grenoble, INSERM U1042, Université Grenoble-Alpes, Grenoble, France; 15 Department of Medicine and Interdepartmental Division of Critical Care Medicine, Sinai Health System, University of Toronto, Toronto, Ontario Canada; 16 Agostino Gemelli University Hospital, Università Cattolica del Sacro Cuore, Rome, Italy; 17 Department of Medical Intensive Care Medicine, University Hospital of Angers, Angers, France; 18 Department of Immunology–Department of Emergencies and Critical Care, University of Oslo, Oslo, Norway; 19 Division of Intensive Care Medicine, Department of Anesthesiology, Intensive Care and Pain Medicine, Helsinki University Hospital, University of Helsinki, Helsinki, Finland; 20 Department of Critical Care, University Medical Center Groningen, Groningen, The Netherlands; 21 Medical-Surgical Intensive Care Unit, Centre Hospitalier de Versailles, Le Chesnay, France; 22 Department of Anesthesiology I, Herlev University Hospital, Herlev, Denmark; 23 Medical ICU, Cochin Hospital, Assistance Publique-Hôpitaux de Paris and University Paris Descartes, Paris, France; 24 Critical Care Department, King’s College Hospital NHS Foundation Trust, London, SE5 9RS UK; 25 Critical Care Center, CHU Lille, School of Medicine, University of Lille, Lille, France; 26 Terapia Intensiva, Hospital Maciel, Montevideo, Uruguay; 27 Department of Medical Intensive Care, Normandie Univ, UNIROUEN, EA-3830, Rouen University Hospital, F-76000 Rouen, France; 28 ICU, Fundação Pio XII - Barretos Cancer Hospital, Barretos, Brazil; 29 Medical Intensive Care Unit, La Source Hospital - CHR Orléans, Orléans, France; 30 Intensive Care Department, University of Southern Denmark, Sønderborg, Denmark; 31 Department of Anaesthesia and Intensive Care, Odense University Hospital, Odense, Denmark; 32 Medical Intensive Care Unit, Hôtel Dieu-HME-University Hospital of Nantes, Nantes, France; 33 Réanimation Polyvalente et Département d’Anesthésie et de Réanimation, Institut Paoli-Calmettes, Marseille, France; 34 ECSTRA Team, Biostatistics and Clinical Epidemiology, UMR 1153, INSERM, Paris Diderot Sorbonne University and Service de Biostatistique et Information Médicale AP-HP, Hôpital Saint-Louis, Saint-Louis, France; 35 Department of Intensive Care Medicine, St. James’s Hospital, St. James’s St, Dublin, Dublin 8 Ireland

**Keywords:** Influenza, Respiratory failure, Sepsis, Critical illness, Immunosuppression

## Abstract

**Background:**

It is unclear whether influenza infection and associated co-infection are associated with patient-important outcomes in critically ill immunocompromised patients with acute respiratory failure.

**Methods:**

Preplanned secondary analysis of EFRAIM, a prospective cohort study of 68 hospitals in 16 countries. We included 1611 patients aged 18 years or older with non-AIDS-related immunocompromise, who were admitted to the ICU with acute hypoxemic respiratory failure. The main exposure of interest was influenza infection status. The primary outcome of interest was all-cause hospital mortality, and secondary outcomes ICU length of stay (LOS) and 90-day mortality.

**Results:**

Influenza infection status was categorized into four groups: patients with influenza alone (*n* = 95, 5.8%), patients with influenza plus pulmonary co-infection (*n* = 58, 3.6%), patients with non-influenza pulmonary infection (*n* = 820, 50.9%), and patients without pulmonary infection (*n* = 638, 39.6%). Influenza infection status was associated with a requirement for intubation and with LOS in ICU (*P* < 0.001). Patients with influenza plus co-infection had the highest rates of intubation and longest ICU LOS. On crude analysis, influenza infection status was associated with ICU mortality (*P* < 0.001) but not hospital mortality (*P* = 0.09). Patients with influenza plus co-infection and patients with non-influenza infection alone had similar ICU mortality (41% and 37% respectively) that was higher than patients with influenza alone or those without infection (33% and 26% respectively). A propensity score-matched analysis did not show a difference in hospital mortality attributable to influenza infection (OR = 1.01, 95%CI 0.90–1.13, *P* = 0.85). Age, severity scores, ARDS, and performance status were all associated with ICU, hospital, and 90-day mortality.

**Conclusions:**

Category of infectious etiology of respiratory failure (influenza, non-influenza, influenza plus co-infection, and non-infectious) was associated with ICU but not hospital mortality. In a propensity score-matched analysis, influenza infection was not associated with the primary outcome of hospital mortality. Overall, influenza infection alone may not be an independent risk factor for hospital mortality in immunosuppressed patients.

**Electronic supplementary material:**

The online version of this article (10.1186/s13054-019-2425-6) contains supplementary material, which is available to authorized users.

## Introduction

Immunosuppressed patients admitted to an intensive care unit (ICU) with acute respiratory failure have a very high risk of mortality [1]. Acute respiratory failure can have various etiologies, but pulmonary infection and its sequelae remain the most frequent precipitants in those that require ICU admission [2, 3]. Among the different infectious agents which cause pulmonary infection in immunocompromised patients, pneumonia caused by influenza viruses has been associated with a particularly high mortality rate [4].

Influenza infection can affect patients during pandemic periods (such as the H1N1 pandemic of 2008/2009) or during seasonal epidemics. Factors associated with the risk of a severe influenza infection during and after pandemic periods have differed [5]. After the first pandemic period in 2008 and 2009, influenza affected particular subgroups, particularly obese and pregnant patients [6, 7]. During the post-pandemic period, immunosuppression was a risk factor for both influenza infection and ICU mortality [8]. Other factors associated with greater severity of influenza infection are age, medical comorbidities, and possibly co-infection. Bacterial pulmonary co-infection has long been described in patients with influenza pneumonia, most commonly with *Staphylococcus aureus*, *Streptococcus pneumoniae*, and *Haemophilus influenzae*. Recognition of influenza infection is important because it allows the implementation of appropriate infection control measures and specific antiviral therapy. Furthermore, it might reduce inappropriate antibacterial administration.

Although bacterial co-infection has been associated with increased mortality during the 2008/2009 pandemic period [9], the impact of the combination of influenza infection and bacterial or fungal co-infection on the outcome of critically ill patients has been a matter of debate [10]. Although influenza seems to be associated with higher mortality rates in immunocompromised patients [11, 12], the fraction of mortality attributable to either influenza infection alone or influenza plus co-infection has not been well defined. Our aim in the current study was to examine the prevalence of influenza infection and co-infection in critically ill immunocompromised patients admitted to the ICU with respiratory failure and to determine whether influenza and associated co-infection were associated with patient-important outcomes in this group.

## Methods

### Study design and setting

The current study was a preplanned secondary analysis of the EFRAIM study, a multinational prospective cohort study in 68 centers in 16 countries. EFRAIM was performed by the Nine-I (Caring for Critically Ill Immunocompromised Patients) study group [13]. The Nine-I group includes critical care physicians from 16 countries who have extensive experience in the management of various groups of critically ill immunocompromised patients. Physician participation was voluntary, without financial incentive. Participating investigators obtained local institutional review board approval in accordance with local ethics regulations.

### Inclusion and exclusion criteria

Eligibility criteria were age ≥ 18 years, acute hypoxemic respiratory failure (PaO_2_ < 60 mmHg or SpO_2_ < 90% on room air, or tachypnea > 30/min, or labored breathing or respiratory distress or dyspnea at rest or cyanosis), need for more than 6 L/min oxygen, respiratory symptom duration less than 72 h, and non-AIDS-related immune deficiency defined as hematologic malignancy or solid tumor (active or in remission for less than 5 years), solid organ transplant, long-term (> 30 days, any dose) or high-dose (> 1 mg/kg/day) steroids, or any immunosuppressive drug taken in a high dosage or for more than 30 days. Exclusion criteria were postoperative acute respiratory failure, admission after a cardiac arrest, ICU admission exclusively to secure bronchoscopy, or refusal of the patient or family to participate in the study.

### Enrolment, data collection, and patient treatment

Participating ICUs enrolled patients from Nov. 5, 2015 to Jul. 1, 2016. Prospective data were collected on patient and disease characteristics, initial oxygenation strategy, acute respiratory failure (ARF) etiology, associated organ dysfunction, and patient outcomes at hospital discharge and at day 90. The case report forms were sent to the coordinating center in Paris for data entry by trained technicians. The study was funded by the Groupe de Recherche en Réanimation Onco-Hématologique (GRRR-OH), an academic non-profit French organization.

All management decisions were performed according to standard local practice in each ICU. Diagnostic strategies to identify the etiology of respiratory failure were based on previous studies by the GRRR-OH (11,18-20,23). ARF etiologies were based on pre-defined criteria in each participating ICU (11,18-20,23). All diagnoses were reviewed by two study investigators (from independent institutions) for coherence and for alignment with accepted definitions. Oxygenation modalities, the use of non-invasive ventilation, high-flow nasal oxygen, or intubation was documented daily. Management of associated organ dysfunction, handling of immunosuppressive drugs, or chemotherapy was decided by a physician according to local and recommended practices. Intubation decisions were left at the discretion of the care team and based on the therapeutic response, clinical status (including SpO_2_, respiratory rate, signs of respiratory distress, and bronchial secretion volume), and patient’s adherence to other oxygenation modalities.

### Exposures, outcomes, and important covariates

The exposure of interest in this prespecified secondary analysis of the EFRAIM study was influenza infection status. Patients were divided into four groups for the purposes of analysis: (1) influenza respiratory tract infection alone, (2) influenza respiratory tract infection plus co-infection, (3) non-influenza respiratory tract infection, and (4) no suspected or confirmed respiratory tract infection. Influenza was diagnosed by the presence of positive reverse transcription polymerase chain reaction (RT-PCR) in immunosuppressed patients admitted to intensive care units (ICUs) by a nasopharyngeal swab as it is the optimal upper respiratory tract specimen collection method for influenza recommended by the CDC [14]. RT-PCR was not performed in all patients but mainly in those in whom influenza infection was suspected.

Pulmonary co-infection was defined as either clinically or microbiologically confirmed bacterial or invasive fungal respiratory infection in patients with influenza RT-PCR-positive respiratory tract infections [15].

The primary study outcome was all-cause hospital mortality. Secondary outcomes included ICU length of stay and 90-day mortality.

Data on important covariates were collected prospectively. SOFA score was recorded at ICU admission [16]. Shock was defined as a need for vasopressors; acute kidney injury (AKI) was defined as a need for renal replacement therapy as decided by the treating physicians.

### Statistical analysis

Continuous variables are reported as medians (interquartile ranges [IQRs]) and categorical variables as proportions. Data management allowed checking for data inconsistencies that were solved by consensus. Comparisons of proportions between the groups were made using the *χ*^2^ test. Comparisons of continuous variables between the groups were made using the Wilcoxon rank-sum test.

A propensity score (PS)-based approach was used to limit the effect of bias on the between-group comparisons of hospital mortality. The propensity score was defined as the probability that a patient with specific baseline characteristics had influenza infection. We developed the PS using a logistic regression model that included all baseline characteristics associated with illness severity [2]: mechanical ventilation, age, Eastern Cooperative Oncology Group (ECOG) performance status, SOFA score at ICU admission, admission within first hours of hospital admission, tobacco use, and underlying disease (hematological or solid tumor or immune disease). To handle the missing values in these confounders, multiple imputations with chained equation were used, where PS for each patient was averaged across 30 completed datasets while PS matching used these averaged scores. We matched individuals based on their PS using a 1:1 matching algorithm without replacement within a caliper of 0.15 standard deviation of the logit of the PS. Final analyses on the matched dataset were then performed using a logistic regression with a random effects model on the paired observations, except for the length of stay which we analyzed with a Cox random effects model. Results are presented as odds ratio (OR) with their 95%CI. Primary analyses were performed on the complete cases, assuming missing completely at random covariates. Sensitivity analyses for such assumptions were performed, based on multiple imputation with chained equation. Details of the sample size calculation for the original EFRAIM study can be found elsewhere [13]. A post hoc power analysis was not considered appropriate for the current secondary analysis. All tests were two sided at the 0.05 significance level. Analyses were performed using R statistical package (online at http://www.R-project.org).

## Results

### Baseline characteristics

Out of 1611 patients (60% men, median age 63 (IQR 54–71)) enrolled in the 68 participating ICUs, 4 exposure groups were defined: patients with influenza respiratory tract infection alone (*n* = 95, 5.8%), patients with influenza plus co-infection (*n* = 58, 3.6%), patients with non-influenza respiratory tract infection (*n* = 820, 50.9%), and patient without suspected or confirmed respiratory tract infection (*n* = 638, 39.6%). We also performed additional analysis on 448 patients negative for influenza and testing not done.

Characteristics of each group are summarized in Table 1. There were no statistically significant differences between the groups regarding general clinical characteristics including age and comorbidities. However, patients with influenza tended to have a higher body mass index and were more frequently admitted to the ICU directly from the emergency department. Patients without any respiratory tract infection were less likely to have hematological disease. SOFA score was higher in patients with influenza and co-infection, mainly driven by higher SOFA respiratory subscores in this group. When excluding patients negative for influenza but with no testing done, only patients with influenza alone were more likely to have solid tumors (Additional file 1: Table S1). Intubation during the ICU stay was higher and shock lower in patients with influenza and co-infection compared to the other groups (Table 2).

**Table 1:** Influenza infection status and baseline characteristics at ICU admission

Baseline characteristics	No infection (*n* = 638)	Infection other than influenza (*n* = 820)	Influenza alone (*n* = 95)	Influenza co-infection (*n* = 58)	*P* value^b^
Age (years), median [IQR]	63 [54–71]	63 [55–71]	65 [54–72]	64 [52–70]	0.80
Gender, male	351 (55)	512 (63)	59 (63)	32 (56)	0.04
Obesity^a^	108 (17)	151 (18)	21 (22)	12 (21)	0.21
Underlying disease					
Hematological disease	311 (49)	436 (53)	60 (63)	30 (51)	0.05
Solid tumor	252 (39)	285 (35)	18 (19)	12 (21)	< 0.001
Solid organ transplantation	51 (9)	75 (10)	7 (8)	9 (16)	0.4
Systemic disease or other ID	102 (16)	133 (16)	25 (26)	18 (31)	0.002
Disease status at ICU admission					
Newly diagnosed	161 (36)	154 (27)	12 (17)	7 (20)	0.0004
Remission	67 (15)	82 (14)	15 (21)	8 (23)	
No remission	62 (14)	68 (12)	3 (4)	6 (17)	
Allogeneic stem cell transplant	56 (9)	82 (10)	9 (9)	5 (9)	0.03
ECOG^c^ ≥ 2 (severely disabled or bedridden)	210 (33)	299 (36)	36 (38)	23 (40)	0.15
Comorbidities					
Cardiac	141 (24)	167 (22)	16 (18)	15 (27)	0.43
COPD	103 (17)	123 (15)	14 (15)	7 (12)	0.80
Kidney	88 (14)	117 (15)	16 (18)	10 (17)	0.75
Diabetes	108 (17)	161 (20)	21 (22)	14 (25)	0.30
Alcohol use disorder	63 (10)	76 (10)	5 (5)	4 (7)	0.48
Tobacco use	199 (33)	228 (29)	21 (23)	12 (21)	0.08
Duration of symptoms before ICU admission (days), median [IQR]	1 [0–4]	1 [0–3]	2 [1–7]	1.5 [1–4]	< 0.001
Admission from emergency department	208 (33)	256 (32)	41 (43)	19 (33)	0.17
Neutropenia at admission	66 (11)	153 (20)	20 (21)	12 (21)	< 0.001

Data are presented as median [IQR] or *N* (%)

^a^Obesity grade I, II and extreme obesity

^b^Chi-squared test of association with three degrees of freedom

^c^Eastern Cooperative Oncology Group (ECOG) performance status score

**Table 2:** Association between influenza infection status, clinical characteristics at day 1, and outcomes

Variables	No infection (*n* = 638)	Infection other than influenza (*n* = 820)	Influenza alone (*n* = 95)	Influenza & co-infection (*n* = 58)	*P* value^a^
At day 1					
Maximum respiratory rate (breaths/min)	30 [24–36]	31 [25–37]	32 [28–36]	32 [26–38]	0.01
Liters/min O_2_	7 [3–15]	8 [5–15]	10 [4–15]	15 [2–15]	0.04
FiO_2_	50 [40–70]	50 [40–80]	50 [50–72]	59 [51–75]	0.03
PaO_2_/FiO_2_ ratio	173 [115–215]	110 [79–173]	113 [110–204]	127 [87–170]	< 0.001
ARDS at day 1	481 (75)	737 (90)	92 (97)	57 (98)	< 0.001
SOFA at ICU admission	6 [4–9]	7 [4–11]	7 [4–10]	8 [6–10]	< 0.001
Respiratory SOFA = 0	103 (17)	94 (12)	9 (10)	1 (2)	< 0.001
Cardiovascular SOFA = 0	334 (53)	341 (42)	34 (37)	25 (43)	< 0.001
Outcome					
Intubation during the ICU stay	357 (56)	57 (60)	530 (65)	47 (81)	< 0.001
Shock	171 (27)	429 (52)	47 (49)	32 (36)	< 0.001
Renal replacement therapy	93 (15)	140 (17)	17 (17)	17 (29)	0.04
Steroids^b^	187 (33)	272 (36)	27 (31)	27 (49)	0.09
ICU-acquired pneumonia	47 (7)	96 (12)	14 (15)	6 (10)	0.01
ICU length of stay (days)	5 [2–10]	7 [3–15]	8 [4–21]	10.5 [5–20]	< 0.001
ICU mortality	165 (26)	302 (37)	31 (33)	24 (41)	< 0.001
Hospital mortality	251 (41)	365 (46)	36 (38)	30 (52)	0.09
Day 90 mortality	291 (45)	410 (50)	38 (40)	32 (55)	0.06

Data are presented as median, IQR, or *N* (%)

^a^Chi-squared test of association with three degrees of freedom

^b^Received steroids in ICU

### Outcomes on crude, propensity score-matched, and multivariate analysis

**Fig. 1:**
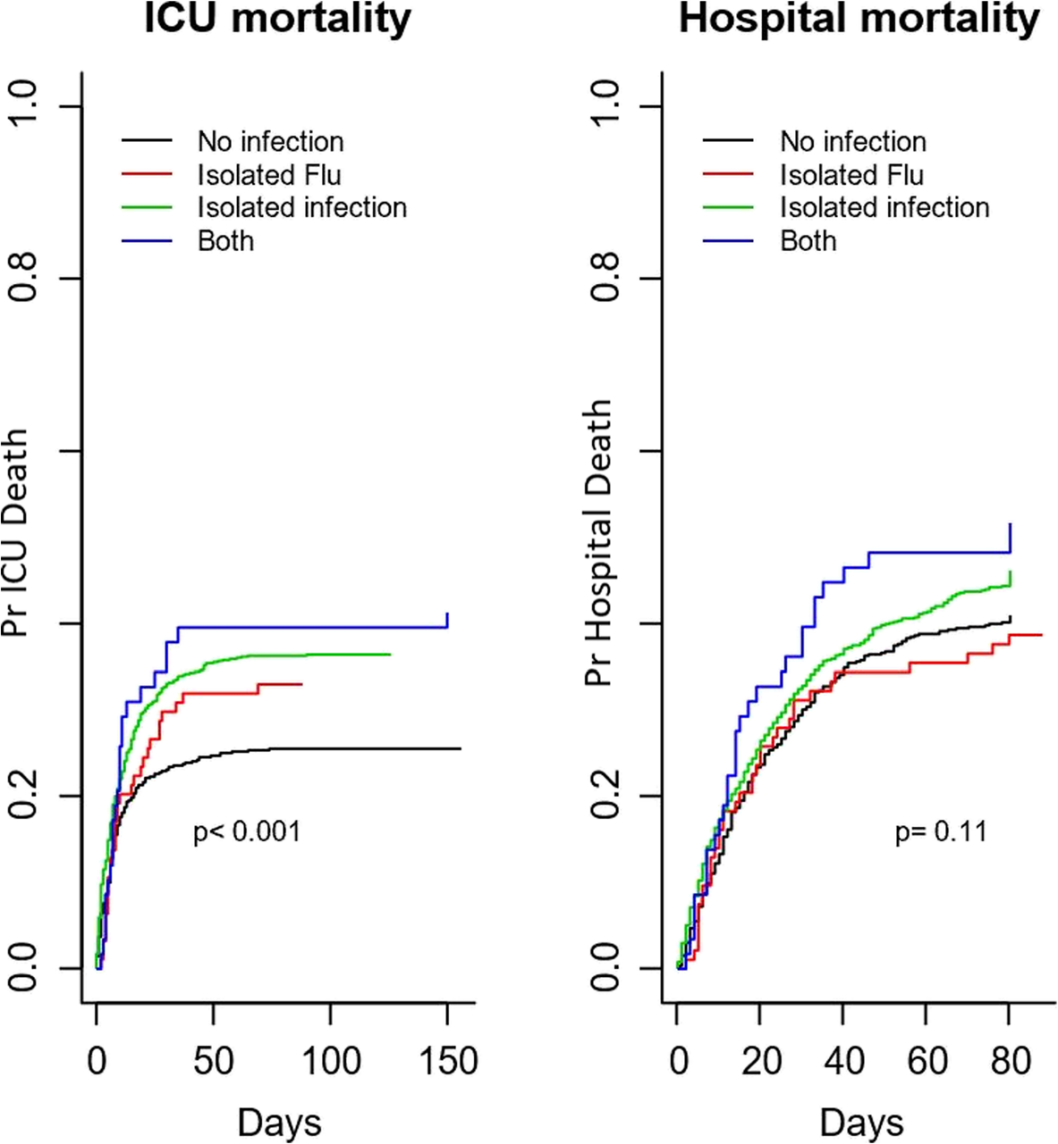
Hospital mortality and influenza infection status. Hospital mortality in the whole cohort according to influenza infection status categorized by four groups: (1) patients with influenza alone, (2) patients with influenza plus co-infections (clinically or microbiologically confirmed bacterial or fungal infection), (3) patients with infections other than influenza infection, and (4) patients without infection. Survival curves were compared using Cox regression

Outcomes in the different exposure groups in the crude analysis are summarized in Table 2. ICU mortality differed between the four groups (*P* < 0.001), with the highest mortality in patients with influenza plus co-infection (41%) and non-influenza infection (37%) and slightly lower mortality in influenza infection alone (33%) and in those without infection (26%). When the analyses were performed, after excluding those patients negative for influenza but with no testing done, similar results were found (Additional file 2: Table S2). Hospital and day 90 mortality showed a trend with the highest mortality in patients with influenza plus co-infection (52%) and non-influenza infection (46%) respectively (Table 2 and Additional file 2: Table S2). Survival curves for ICU and hospital stay for the four groups are presented in Fig. 1. Hospital survival did not differ by group (*P* = 0.11).

**Fig. 2:**
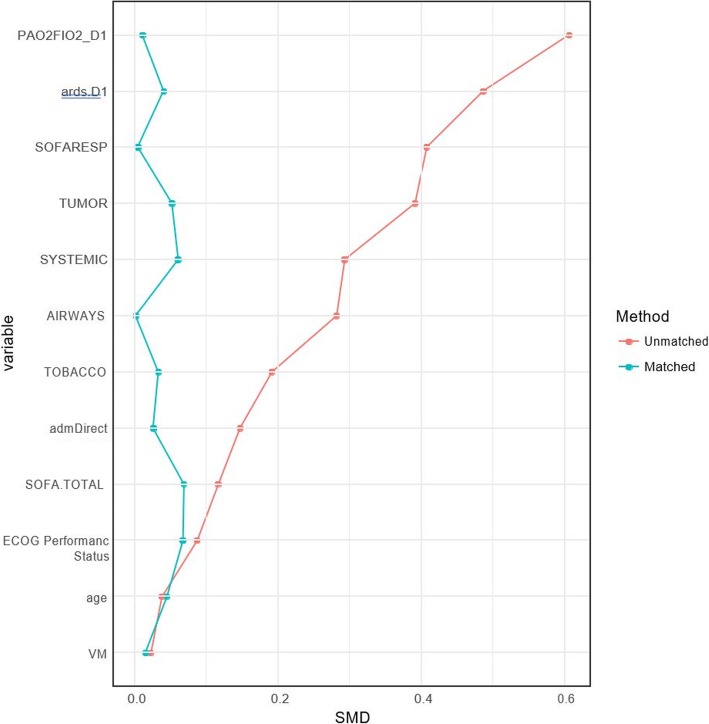
Imbalances in confounders of mortality by influenza infection status before and after propensity score matching. Based on the matched sample, there was no evidence of any difference in hospital mortality across groups (OR = 1.01, 95%CI 0.90–1.13, *p* = 0.85). We developed a propensity score (PS) logistic model to have flu then matched the individuals on the basis of their PS using a 1:1 matching algorithm without replacement within a caliper of 0.15 standard deviation of the logit of the propensity score. To handle missing values in confounders, multiple imputation with chained equation was used for the PS model, where propensity score for each patient was averaged across 30 completed datasets while propensity score matching used these averaged scores to estimate the treatment effect. Only 1 patient with influenza could not be matched. Imbalances in confounders were reduced after matching

For the propensity score-matched analysis, 152 patients with influenza were matched. One patient with influenza could not be matched and was excluded from the analysis. Imbalances in confounders were reduced after matching (Fig. 2). In the matched sample, there was no difference in hospital mortality attributable to influenza infection (OR = 1.01, 95%CI 0.90–1.13, *P* = 0.85).

Table 3 shows the results of multivariate analysis after multiple imputation by chained equations. The following factors were associated with hospital mortality: age, direct ICU admission, severity manifested by SOFA score, diagnosis of ARDS, and performance status, with the latter two demonstrating the strongest association with OR of hospital mortality of 1.53 and 1.44 respectively. None of the mechanism and/or type of immunosuppression was found as an independent risk factor for hospital mortality. When the analysis was performed in patients negative for influenza and the test not done, similar results were found (Table 3).

**Table 3:** Multivariate analysis of factors associated with hospital mortality after multiple imputations

Variable	Assuming non-tested = negative		Excluding non-tested	
	OR	*P* value	OR	*P* value
No infection	1.00		1.00	
Influenza alone	0.79 (0.49–1.27)	0.33	0.96 (0.51–1.78)	0.89
Infection other than influenza	1.02 (0.81–1.29)	0.85	1.28 (0.85–1.93)	0.23
Influenza co-infection	1.21 (0.68–2.15)	0.51	1.94 (0.84–3.72)	0.09
Age	1.01 (1.003–1.019)	0.0031	1.01 (1.003–1.019)	0.0051
Direct admission	0.72 (0.57–0.91)	0.0061	0.73 (0.53–0.99)	0.0042
SOFA score	1.14 (1.107–1.171)	< 0.0001	1.15 (1.11–1.19)	< 0.0001
ARDS	1.53 (1.12–2.10)	0.0084	1.57 (0.97–2.53)	0.065
ECOG	1.44 (1.29–1.61)	< 0.0001	1.46 (1.27–1.67)	< 0.0001

Mechanism and/or type of immunosuppression was not found as an independent risk factor/s for hospital mortality

## Discussion

In summary, our multinational observational study analyzed 1611 immunosuppressed patients from 68 centers and found that if a critically ill immunosuppressed patient is infected with influenza, the outcome depends on the immunosuppression (independently of the mechanism and/or type of immunosuppression) rather than influenza infection. We found that independent risk factors for hospital mortality were age, organ dysfunction severity, direct admission to the ICU, and especially a diagnosis of ARDS and performance status. Influenza plus co-infection with bacterial or fungal pathogens was associated with the highest ICU mortality rate in our study. We did not observe a statistically significant association between influenza infection status and hospital mortality, our primary outcome, in either crude or propensity score-matched analyses.

Influenza is a risk factor for acute respiratory failure in immunosuppressed patients. However, its role as an independent risk factor for mortality in such a population has been questioned [9]. The number of immunosuppressed patients hospitalized with influenza has increased in recent years, and we showed that while influenza alone may not increase mortality, influenza plus co-infection may be associated with higher ICU mortality. It might therefore be argued that empiric antibiotic treatment for co-infection in such patients should be considered until the possibility of co-infection has been confidently ruled out. To facilitate earlier detection of co-infection, Rodriguez et al. [17] recently described that a low level of procalcitonin (PCT) has a high negative predictive value (94%). However, clinicians may not be willing to tolerate even a low probability of untreated pulmonary co-infection in light of our observation that this category was associated with higher ICU mortality and length of stay.

In our cohort, two important factors stood out as independent risk factors for death: the need for intubation during ICU stay and ARDS. While both features were associated with increased mortality in any immunosuppressed patient, the mortality rate approached 100% when this occurred in patients with influenza and co-infection. Due to a known protective effect on mortality of direct admission from the emergency department to the ICU, it might be hypothesized that earlier assessment for severity and therefore earlier ICU admission may improve the outcomes [18]. In a large population of patients with influenza, Álvarez-Lerma et al. [19] observed that ICU mortality was significantly higher among patients with late diagnosis as compared with early diagnosis (26.9% vs 17.1%, *P* < 0.001). Diagnostic delay was one independent risk factor for mortality (OR = 1.36, 95%CI 1.03–1.81, *P* < 0.001).

A common diagnostic challenge in immunosuppressed patients is the lack of clinical symptoms when developing infections. In other words, an immunocompromised host is a patient who does not have the ability to respond normally to an infection due to an impaired or weakened immune system. This has been well reported in cases of bacterial or fungal infections, but little is known in the case of viral infections [20]. In our cohort, we found that patients with influenza were actually more likely to have a longer duration of symptoms prior to ICU admission.

Hypoxemia is a common clinical feature of patients with influenza, especially in immunosuppressed hosts. In a recent report from two cancer centers describing the outcomes in patients with hematological malignancies and influenza infection, severe hypoxemia was an independent risk factor (OR 5.87, 1.12–30.77) for 60-day mortality [21]. Similarly, hypoxemia was clearly a signal of illness severity in our study. In patients not intubated at admission to the ICU, oxygen requirements and ICU mortality rates were greatest in those with influenza plus co-infection.

Co-infection has previously been reported as an independent risk factor for poor outcome in patients with influenza [9]. In our cohort, patients with co-infection were less likely to be cancer patients (have a hematological disease or solid tumor) but were more likely to have newly diagnosed immunosuppressive systemic disease or have poorer functional capacity. In this population, the criteria that suggest co-infection and therefore higher severity may be higher oxygen requirements, greater tachypnea and work of breathing, and higher rates of mechanical ventilation.

Systemic immune mechanisms play a key role in the development of co-infection based on the complexity of the interaction of the host and the viral and bacterial pathogens. Several studies have been performed to determine the point prevalence of bacterial co-infection in influenza patients [9, 22–24]. In our cohort, almost half of the patients with co-infection received steroids. The use of steroids has been controversial and is currently not recommended in patients with influenza [25]. This is particularly relevant to our studied population because many patients were already receiving corticosteroid therapy for their primary disease. It appears plausible that steroids were given as a stress response treatment in patients that were using longer-term steroids and not as a treatment for influenza per se. Importantly, we did not find steroids to be a risk factor for hospital mortality.

Some limitations should be mentioned. Vaccination status and information on antiviral regimen, dose, duration, and delay in the start of therapy were not collected. Similar limitations apply to the determination of co-infection, which also could have led to misclassification error and bias. The sample contained primarily patients with underlying hematological disease. Other subgroups of immunocompromised patients (particularly patients with lung transplant) may be underrepresented which may limit generalizability. Additionally, we did not completely account for the effect of the type of immunosuppressive regimen in the adjusted analysis. The propensity score analysis aims at controlling for confounders, including those variables associated with the immunodeficiency that may affect the outcome. Nevertheless, one cannot assume that all confounders—possibly even not observed—have been taken into account and that there may be residual confounders.

## Conclusion

In summary age, severity score, ARDS, and performance status were all independent risk factors for ICU, hospital, and 90-day mortality in immunosuppressed patients admitted to the ICU for acute hypoxemic respiratory failure. The main aim in this paper was to determine if influenza alone or co-infection played a role in the mortality in ICU patients. Category of infectious etiology of respiratory failure (influenza, non-influenza, influenza plus co-infection, and non-infectious) was associated with ICU but not hospital mortality. In a propensity score-matched analysis, influenza infection was not associated with the primary outcome of hospital mortality. Overall, influenza infection alone is not an independent risk factor for hospital mortality in immunosuppressed patients.

## Additional files

Additional file 1: **Table S1.** Influenza infection status and baseline characteristics at ICU admission. Group no infection performed excluding 448 patients negative for influenza and testing not done. (DOCX 18 kb)

Additional file 2: **Table S2.** Association between influenza infection status, clinical characteristics at day 1, and outcomes. (DOCX 36 kb)
