# Supplementary material for: Influenza and associated co-infections in critically ill immunosuppressed patients
Source: Crit Care. 2019 May 2;23:152. doi: 10.1186/s13054-019-2425-6 (PMC6498695; doi:10.1186/s13054-019-2425-6)
Supplement: Supplementary file 1 — Table S1. Influenza infection status and baseline characteristics at ICU admission. Group no infection performed excluding 448 patients negative for influenza and testing not done. (DOCX 18 kb) [file 13054_2019_2425_MOESM1_ESM.docx]

**Table S1 : Influenza infection status and baseline characteristics at ICU admission. Group no infection performed excluding 448 patients negative for influenza and testing not done.**

| Baseline characteristics | No infection (n=190) | Infection other than influenza  (n=820) | Influenza alone (n= 95) | Influenza co- infection  (n= 58) | P  Value^b^ |
| --- | --- | --- | --- | --- | --- |
| Age (year) median, [IQR] | 64 [54-72] | 63 [55-71] | 65 [54-72] | 64 [52-70] | 0.75 |
| Gender, male | 98 (52) | 512 (63) | 59 (63) | 32 (56) | 0.04 |
| Obesity^a^ Underlying disease  Hematological disease | 50 (29)  104 (55) | 151 (18)  436 (53) | 21 (22)  60 (63) | 12 (21)  30 (51) | 0.13  0.31 |
| Solid tumor  Solid organ transplantation Systemic disease or other ID | 65 (34)  10 (6)  36 (19) | 285 (35)  75 (10)  133 (16) | 18 (19)  7 (8)  25 (26) | 12 (21)  9 (16)  18 (31) | 0.003  0.11  0.005 |
| Disease status at ICU admission |  |  |  |  |  |
| Newly diagnosed | 41 (31) | 154 (27) | 12 (17) | 7 (20) | 0.014 |
| Remission | 19 (14) | 82 (14) | 15 (21) | 8 (23) |  |
| No remission | 22 (16) | 68 (12) | 3 (4) | 6 (17) |  |
| Allogeneic stem cell transplant | 25 (13) | 82 (10) | 9 (9) | 5 (9) | 0.003 |
| ECOG^c^ ≥2 (severely disabled or bedridden) | 60 (31) | 299 (36) | 36 (38) | 23 (40) | 0.30 |
| Comorbidities |  |  |  |  |  |
| Cardiac | 40 (22) | 167 (22) | 16 (18) | 15 (27) | 0.65 |
| COPD | 40 (22) | 123 (15) | 14 (15) | 7 (12) | 0.16 |
| Kidney | 29 (15) | 117 (15) | 16 (18) | 10 (17) | 0.84 |
| Diabetes | 31 (17) | 161 (20) | 21 (22) | 14 (25) | 0.47 |
| Alcohol use disorder | 11 (6) | 76 (10) | 5 (5) | 4 (7) | 0.28 |
| Tobacco use | 66 (37) | 228 (29) | 21 (23) | 12 (21) | 0.037 |
| Duration of symptoms before ICU admission (days), median, [IQR] | 1 [0-4] | 1 [0-3] | 2 [1-7] | 1.5 [1-4] | <0.001 |
| Admission from emergency department | 37 (21) | 256 (32) | 41 (43) | 19 (33) | 0.09 |
| Neutropenia at admission | 25 (14) | 153 (20) | 20 (21) | 12 (21) | <0.001 |

**Data are presented as median [IQR], or N (%)**

**a obesity grade I, II and extreme obesity; b Chi-squared test of association with three degrees of freedom; c Eastern Cooperative Oncology Group (ECOG) performance status score**
