# Supplementary material for: Influenza and associated co-infections in critically ill immunosuppressed patients
Source: Crit Care. 2019 May 2;23:152. doi: 10.1186/s13054-019-2425-6 (PMC6498695; doi:10.1186/s13054-019-2425-6)
Supplement: Supplementary file 2 — Table S2. Association between influenza infection status, clinical characteristics at day 1, and outcomes. (DOCX 36 kb) [file 13054_2019_2425_MOESM2_ESM.docx]

**Table S2: Association between influenza infection status, clinical characteristics at Day 1 and outcomes**

| Variables | | | No infection (n=190) | | | Infection other than influenza  (n=820) | | | Influenza alone (n= 95) | | | Influenza & co- infection  (n= 58) | | | P Value^a^ | | |
| --- | --- | --- | --- | --- | --- | --- | --- | --- | --- | --- | --- | --- | --- | --- | --- | --- | --- |
| **At D1** | | |  | | |  | | |  | | |  | | |  | | |
| Maximum respiratory rate (breaths/min) | | | 30 [25-36] | | | 31 [25-37] | | | 32 [28-36] | | | 32 [26-38] | | | 0.007 | | |
| Litres/min O2 FiO2  PaO2/ FiO2 ratio | | | 7 [5-8]  50 [40-60]  173 [104-206] | | | 8 [5-15]  50 [40-80]  110 [79-173] | | | 10 [4-15]  50 [50-72]  113 [110-204] | | | 15 [2-15]  59 [51-75]  127 [87-170] | | | 0.04  0.002  <0.001 | | |
| ARDS at day 1 | | 150 (79) | | | 737 (90) | | | 92 (97) | | | 57 (98) | | | <0.001 | | |  |
| SOFA at ICU admission | | 7 [4-10] | | | 7 [4-11] | | | 7 [4-10] | | | 8 [6-10] | | | 0.32 | | |  |
| Respiratory SOFA =0 | | 25 (14) | | | 94 (12) | | | 9 (10) | | | 1 (2) | | | <0.001 | | |  |
| Cardiovascular SOFA =0 | | 89 (47) | | | 341 (42) | | | 34 (37) | | | 25 (43) | | | <0.001 | | |  |
| **Outcome** | |  | | |  | | |  | | |  | | |  | | |  |
| Intubation during the ICU stay | | 117 (61) | | | 57 (60) | | | 530 (65) | | | 47 (81) | | | 0.037 | | |  |
| Shock | | 68 (35) | | | 429 (52) | | | 47 (49) | | | 32 (36) | | | <0.001 | | |  |
| Renal replacement therapy | | 36 (16) | | | 140 (17) | | | 17 (17) | | | 17 (29) | | | 0.15 | | |  |
| Steroids^b^ | | 59 (34) | | | 272 (36) | | | 27 (31) | | | 27 (49) | | | 0.17 | | |  |
| ICU acquired pneumonia | | 18 (9) | | | 96 (12) | | | 14 (15) | | | 6 (10) | | | 0.64 | | |  |
| ICU length of stay (day) | | 6 [3-12] | | | 7 [3-15] | | | 8 [4-21] | | | 10.5 [5-20] | | | 0.006 | | |  |
| ICU mortality | | 55 (29) | | | 302 (37) | | | 31 (33) | | | 24 (41) | | | 0.14 | | |  |
| Hospital Mortality | | 72 (40) | | | 365 (46) | | | 36 (38) | | | 30 (52) | | | 0.15 | | |  |
| Day 90 mortality | | 87 (45) | | | 410 ( 50) | | | 38 ( 40) | | | 32 (55) | | | 0.06 | | |  |
| Data are presented as median, IQR or N(%)  a b Chi-squared test of association with three degrees of freedom ; b= received steroids in ICU | | | | |  | | |  | | |  | | |  | | |  |
|  | | |  | | |  | | |  | | |  | | |  | | |

| ARDS at day 1 | 150 (79) | 737 (90) | 92 (97) | 57 (98) | <0.001 |
| --- | --- | --- | --- | --- | --- |
| SOFA at ICU admission | 7 [4-10] | 7 [4-11] | 7 [4-10] | 8 [6-10] | 0.32 |
| Respiratory SOFA =0 | 25 (14) | 94 (12) | 9 (10) | 1 (2) | <0.001 |
| Cardiovascular SOFA =0 | 89 (47) | 341 (42) | 34 (37) | 25 (43) | <0.001 |
| **Outcome** |  |  |  |  |  |
| Intubation during the ICU stay | 117 (61) | 57 (60) | 530 (65) | 47 (81) | 0.037 |
| Shock | 68 (35) | 429 (52) | 47 (49) | 32 (36) | <0.001 |
| Renal replacement therapy | 36 (16) | 140 (17) | 17 (17) | 17 (29) | 0.15 |
| Steroids^b^ | 59 (34) | 272 (36) | 27 (31) | 27 (49) | 0.17 |
| ICU acquired pneumonia | 18 (9) | 96 (12) | 14 (15) | 6 (10) | 0.64 |
| ICU length of stay (day) | 6 [3-12] | 7 [3-15] | 8 [4-21] | 10.5 [5-20] | 0.006 |
| ICU mortality | 55 (29) | 302 (37) | 31 (33) | 24 (41) | 0.14 |
| Hospital Mortality | 72 (40) | 365 (46) | 36 (38) | 30 (52) | 0.15 |
| Day 90 mortality | 87 (45) | 410 ( 50) | 38 ( 40) | 32 (55) | 0.06 |
| Data are presented as median, IQR or N(%)  a Chi-Squared test of association; b= received steroids in ICU | |  |  |  |  |

Table 2: Association between influenza infection status, clinical characteristics at Day 1 and outcomes

Table 3 Multivariate analysis of factors associated with hospital mortality after multiple imputations

| **Assuming non tested=negative** | | | **Excluding non tested** | |
| --- | --- | --- | --- | --- |
|  | | |  | |
| **Variable** | **OR** | **P value** | **OR** | **P value** |
| No infection | 1.00 |  | 1.00 |  |
| Influenza alone | 0.79 (0.49-1.27) | 0.33 | 0.96 (0.51-1.78) | 0.89 |
| Infection other than influenza | 1.02 (0.81-1.29) | 0.85 | 1.28 (0.85-1.93) | 0.23 |
| Influenza co-infection | 1.21 (0.68-2.15) | 0.51 | 1.94 (0.84-3.72) | 0.09 |
| Age | 1.01 (1.003-1.019) | 0.0031 | 1.01 (1.003-1.019) | 0.0051 |
| Direct Admission | 0.72 (0.57-0.91) | 0.0061 | 0.73 (0.53-0.99) | 0.0042 |
| SOFA score | 1.14 (1.107-1.171) | <0.0001 | 1.15 (1.11-1.19) | <0.0001 |
| ARDS | 1.53 (1.12-2.10) | 0.0084 | 1.57 (0.97-2.53) | 0.065 |
| ECOG | 1.44 (1.29-1.61) | <0.0001 | 1.46 (1.27-1.67) | <0.0001 |
